# Complement 3 mediates periodontal destruction in patients with type 2 diabetes by regulating macrophage polarization in periodontal tissues

**DOI:** 10.1111/cpr.12886

**Published:** 2020-08-14

**Authors:** Ye Li, Xinxin Wang, Saisai Wang, Chunhui Zhu, Jing Guo, Ke Li, Ang Li

**Affiliations:** ^1^ Key Laboratory of Shaanxi Province for Craniofacial Precision Medicine Research College of Stomatology Xi’an Jiaotong University Xi’an China; ^2^ Department of Periodontology College of Stomatology Xi’an Jiaotong University Xi’an China; ^3^ Core Research Laboratory The Second Affiliated Hospital School of Medicine Xi'an Jiaotong University Xi’an China

## Abstract

**Objectives:**

Diabetes aggravates the risk and severity of periodontitis, but the specific mechanism remains confused. Complement 3 (C3) is closely related to complications of type 2 diabetes (T2DM). In the present study, we concentrated on whether C3 mediates the development of periodontitis in T2DM.

**Materials and Methods:**

Levels of C3 in blood and gingival crevicular fluid (GCF) of patients were measured first. A C3‐knockout diabetic mouse model was established, real‐time PCR, Western blotting and histological investigation were performed to evaluate the progress of periodontitis. Microcomputed tomography (micro‐CT) and TRAP staining were performed to detect alveolar bone resorption. Immunofluorescence was performed to detect polarization of macrophages.

**Results:**

Our data showed that C3 levels were elevated in the blood and GCF of T2DM patients compared with non‐diabetic individuals. Increased C3 was closely related to the upregulation of inflammatory cytokines including interleukin (IL)‐1, IL‐6 and tumour necrosis factor‐alpha (TNF‐α), as well as the decline of the bone volume density (BMD) and bone volume over total volume (BV/TV) of the alveolar bones in diabetic mice. The deletion of C3 inhibited inflammatory cytokines and rescued the decreased BMD and BV/TV of the alveolar bones. C3‐mediated polarization of macrophages was responsible for the damage.

**Conclusion:**

T2DM‐related upregulation of C3 contributes to the development of periodontitis by promoting macrophages M1 polarization and inhibiting M2 polarization, triggering a pro‐inflammatory effect on periodontal tissues.

## Graphical abstract

T2DM led to an increase in C3 levels, which affected the macrophages in periodontal tissues by promoting macrophages to polarize to M1 and inhibiting M2 polarization, thus triggering a pro‐inflammatory effect on periodontal tissues. C3‐related osteoclasts increase potentially promoted alveolar bone resorption.

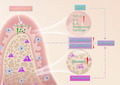

## 1 INTRODUCTION

Type 2 diabetes (T2DM), also known as adult‐onset diabetes, accounts for more than 90% of diabetic patients.^1^ It is a group of chronic metabolic diseases characterized by high blood glucose that causes chronic damage and dysfunction in different tissues, especially the eyes, kidneys, heart, blood vessels and nerves.^2^, ^3^ Periodontitis is known as the sixth complication of diabetes.^4^ Diabetes increases the risk and severity of periodontitis, but the specific mechanism remains unclear.^5^, ^6^, ^7^

The complement system is closely related to the development of diabetes complications.^8^ A large number of complement components, activated fragments and end product membrane attack complex (MAC) deposits are found in the glomerular basement membrane, retinal vascular layer and neural tissues of patients with chronic complications of diabetes.^9^, ^10^, ^11^ Complement 3 (C3) is a key molecule of the complement system.^12^, ^13^ C3 activation was detected in T2DM.^14^ Epidemiological and experimental studies have jointly suggested that C3 is closely related to vascular complications of diabetes and exacerbates diabetic nephropathy, retinopathy and neurological disease.^15^, ^16^, ^17^ In addition, C3 was hyper‐activated in periodontitis. Deletion of C3 has been shown to effectively inhibit the destruction of periodontal tissue and alveolar bone resorption in a periodontitis mouse model.^18^, ^19^ Thereafter, further investigation into whether C3 mediates the development of periodontitis in T2DM patients is necessary.

The purpose of this study was to clarify the role of C3 in the progression of T2DM‐related periodontitis. In addition, preliminary mechanism was explored. In the present study, the levels of C3 in blood and gingival crevicular fluid (GCF) of patients were measured first, and a C3‐knockout diabetic mouse model was established to study the roles of C3 in the progression of T2DM‐related periodontitis.

## 2 MATERIALS AND METHODS

### 2.1 Case inclusion

A total of 17 T2DM patients in the Department of Endocrinology, the Second Affiliated Hospital of Xi'an Jiaotong University, Shaanxi Province, China were included in this study. T2DM was diagnosed according to the standard of the American Diabetes Association published in 2018.^20^ Non‐diabetic individuals (n = 19), patients with periodontitis (n = 17) and diabetic periodontitis patients (n = 14) were enrolled from the outpatient clinic of the Stomatological Hospital (College) of Xi'an Jiaotong University.

The inclusion criteria of T2DM: patients diagnosed with T2DM for more than six months and in stable condition; good periodontal conditions without gingivitis or periodontitis; no antibiotic or immunosuppressant treatment in the past three months; not pregnant; and no acute infections or allergies. The inclusion criteria of the non‐diabetic patients: individuals with normal blood glucose without type‐1 diabetes or T2DM; who had good periodontal conditions without gingivitis or periodontitis; who had no systemic diseases; who did not consume antibiotics or immunosuppressants in the past three months; who were not pregnant; and who had no acute infection or allergy. The inclusion criteria of periodontitis: patients diagnosed with periodontitis without any periodontal treatments in the last 6 months; who with normal blood glucose without type‐1 diabetes or T2DM; no antibiotic or immunosuppressant treatment in the past three months; not pregnant; and no acute infections or allergies. The inclusion criteria of diabetic periodontitis: patients diagnosed with T2DM for more than six months and in stable condition; patients diagnosed with periodontitis without any periodontal treatments in the last 6 months; no antibiotic or immunosuppressant treatment in the past three months; not pregnant; and no acute infections or allergies.

All included patients signed the written informed consent and agreed to the test of C3 content in blood and GCF and the routine periodontal examinations. All operations were performed in accordance with the relevant ethical regulations of Xi'an Jiaotong University and passed the ethical review of the Secondary Affiliated Hospital of Xi'an Jiaotong University and the Stomatological Hospital (College) of Xi'an Jiaotong University.

### 2.2 Sample collection and clinical evaluation

Six millilitre of peripheral blood was collected from all patients with empty stomach in the morning, serum was centrifuged at 4°C, 3000 r/min, 15 minutes, and then stored at −70°C. Filter paper (Whatman) was prepared into 2 mm × 8 mm size. Bilateral maxillary and mandible molars (The third molars were not included) were tested at mesial, distal and buccal positions. For GCF collection, the filter paper was inserted into the sulcus for 30 seconds and washed by centrifugation with 1% PBS. Bleeding on Probing (BOP), gingival index (GI), probing depth (PD) and tooth mobility (Mob) were evaluated for each patient.

### 2.3 Animals

C57BL/6 male mouse weighing ~23 g (n = 40) was used in the present study. All mice were housed under standard conditions with a 12 hour light/dark cycle in a specific‐pathogen‐free (SPF) facility. Mice were randomly divided into four groups. The control group was fed with basic food for 4 weeks before 4 weeks of injection with normal saline (n = 10). The STZ group was fed with basic food for 4 weeks before 4 weeks of injection with streptozotocin (STZ, 60 mg/kg) (n = 10). Four weeks of high‐sugar and high‐fat food followed by 4 weeks of normal saline injection were performed as HF/HS diet group (n = 10). Four weeks of high‐sugar and high‐fat food followed by 4 weeks of low dose (60 mg/kg) STZ intraperitoneal injection were performed to simulate T2DM (n = 10). Fasting glucose was detected using a glucose analyzer, and fasting glucose ≥ 16.7 mmol/L was determined as diabetic status.^21^ SPF grade 4‐week‐old C3−/− mice (n = 10) and corresponding C3+/+ wild‐type (WT) controls (n = 10) were provided by professor Ke Li (Core Research Laboratory, the Second Affiliated Hospital, School of Medicine, Xi'an Jiaotong University). Same protocol of T2DM simulation was performed with both WT and KO mice.

### 2.4 Isolation of total RNA and RT‐PCR

Gingiva was harvested from the maxillary second molars and stored at −70°C for real‐time PCR. Total RNA was isolated using the Trizol reagent (Invitrogen) according to the manufacturer's instructions and diluted in 30 μL of RNase‐free water. Equal amounts of total RNA (2 μg) were reverse transcribed with a First Strand cDNA Synthesis Kit (Fermentas). Gene expression was analysed using an iQ5 (Bio‐Rad) with SYBR^®^ Premix Ex Taq™ II (TaKaRa). Sequences of the primers are provided in Table 1.

**Table 1:** Primer sequences

Gene	ID	Primer sequence (5′‐3′) forward primer reverse primer	Product (bp)
β‐actin	NM_007393.5	CACGATGGAGGGGCCGGACTCATC TAAAGACCTCTATGCCAACACAGT	241
IL‐6	NM_001314054.1	GCCTTCTTGGGACTGATGCT TGTGACTCCAGCTTATCTCTTGG	448
IL‐1	NM_001146087.1	GCCAGAGGCAGTCCTTTCAA GCTTGCTCCTCCAAAATGCC	95
TNF‐α	NM_001278601.1	ACCCTCACACTCACAAACCA ACCCTGAGCCATAATCCCCT	564
C3	NM_009778.3	GCTTCAGGGTCCCAGCTACT GGGCAGTAGGTTGTTGTCCA	468

### 2.5 Western blotting

Rabbit Anti‐C3 antibody (1:500, ab200999) and β‐actin (1:2000, ab179467) antibody (Abcam) were used. Protocols of Western blotting were used as previously described.^22^ Three independent repeated experiments were performed by different operators.

### 2.6 Histological examinations

Maxillary bones were excised and immediately fixed in 4% paraformaldehyde neutral buffer solution for 48 hours. Then, the maxillary specimens were decalcified with 10% EDTA at room temperature for 7 days until the alveolar bone could be easily penetrated followed by conventional dehydration and paraffin embedding. Immunohistochemical detection of C3 and RANKL as well as HE staining was performed in each group of samples. Examination and analysis were performed in blind. For immunohistochemical detection, 5 μm sections were prepared. Deparaffinized sections were treated with methanol containing 3% hydrogen peroxide before conducting antigen retrieval using a microwave oven at 95°C for 5 minutes, and cooling at room temperature for 15 minutes for two times. After washing with phosphate‐buffered saline (PBS), 5% bovine serum albumin was applied for 10 minutes. The sections were incubated with antibody 16‐18 hours overnight at 4°C. Then, after washing by PBS, a biotin‐conjugated secondary antibody was applied for 30 minutes. DAB chromogenic agent kit (Boster) was used to develop colour and the samples were counterstained with haematoxylin. Antibodies used were Rabbit Anti‐C3 antibody (1:200, Abcam, ab200999) and Rabbit Anti‐RANKL antibody (1:200, Abcam, ab216484).

### 2.7 Micro‐computed (Micro‐CT)

Bilateral maxillary was fixed in 4% paraformaldehyde. Tissues were scanned on a micro‐CT system. The parameters are set as follows: 70 kV, 114 μA, 12 μm resolution; Root bifurcation area of second molar was defined as the volume of interest (VOI). Thirty slices prior to and after the identified furcation slice were added to generate a VOI. The distance between cemento‐enamel junction and the alveolar bone crest (CEJ‐ABC) was assessed. Bone volume density (BMD) and bone volume over total volume (BV/TV) were analysed.

### 2.8 Tartrate‐resistant acid phosphatase (TRAP)

TRAP staining was performed by using Acid Phosphatase, Leukocyte (TRAP) Kit (Sigma) according to the manufacturer's instructions. Briefly, slices were deparaffinized and rinsed with deionized water. TRAP staining was performed at 37°C in the dark for 1 hour. Nuclei were stained with haematoxylin for 2 minutes.

### 2.9 Immunofluorescence

Detailed methods are described as previously.^22^ Briefly, 5 μm sections were prepared. Slices were deparaffinized and incubated with 5% bovine serum albumin for 30 minutes. Afterwards, samples were incubated with primary antibody at 4°C overnight. After washing with PBS, the sections were incubated for with secondary antibody for 2 hours. Fields of connective tissue adjacent to the junctional epithelium as well as alveolar bone were selected from each sample to calculate the number of positive cells. Primary antibodies used were as follows: Rat Anti‐CD68 antibody (1:100, Abcam, ab53444) and Rabbit Anti‐CD206 antibody (1:200, Abcam, ab64693). Secondary antibodies used were as follows: Goat Anti‐Rat IgG H&L (1:1000, Abcam, ab150157) and Goat Anti‐Rabbit IgG H&L (1:2000, Abcam, ab150079).

### 2.10 Statistical analysis

The quantitative data shown in the figures are presented as the mean ± SD. One‐way analysis of variance (ANOVA) and Student's *t* test were performed to analyse differences among or between groups using SPSS 16.0 software (SPSS, Inc). In all analyses, a *P* value < .05 was taken as the level of significance.

## 3 RESULTS

### 3.1 C3 levels were increased in the blood and GCF of T2DM patients

**Figure 1:**
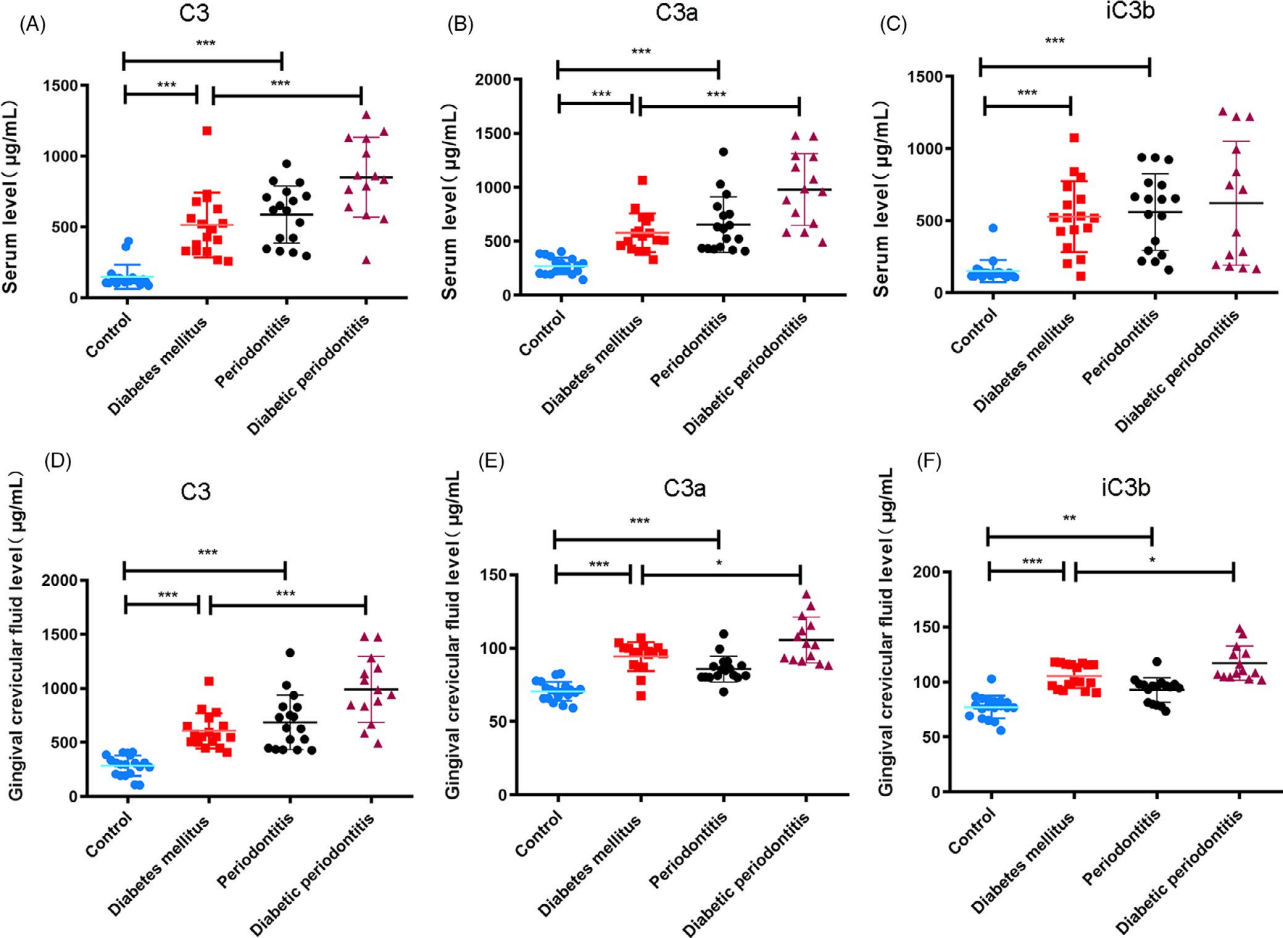
C3 levels were increased in the blood and GCF of T2DM patients. A, The blood C3 level of the non‐diabetic individuals, T2DM patients, patients with periodontitis and diabetic periodontitis patients. ***P < .001. B, The blood C3a levels in the four groups. ***P < .001. C, iC3b levels in the blood of four groups. ***P < .001. D, C3 levels in the GCF of non‐diabetic individuals, T2DM patients, patients with periodontitis and diabetic periodontitis patients. ***P < .001. E, The GCF C3a level of the four groups. *P < .05, ***P < .001. F, The GCF iC3b level of the four groups. *P < .05, **P < .01, ***P < .001

In this study, we compared the levels of C3 and its fragments in the blood of non‐diabetic individuals, T2DM patients, periodontitis patients and diabetic periodontitis patients. Table 2 shows the age and sex of the participants of this study. As shown in Figure 1A‐C, the blood C3, C3a and iC3b of the T2DM patients were elevated compared with non‐diabetic individuals. What's more, the concentration of C3 and C3a was higher in diabetic periodontitis patients compared with T2DM patients. Further measurement of the C3 levels in the GCF of the four groups showed that T2DM patients had higher C3, C3a and iC3b levels than non‐diabetic individuals. In addition, their levels in diabetic periodontitis patients were elevated compared with T2DM patients (Figure 1D‐F).

**Table 2:** Sex and age of the participants

Group	Gender		Age
	Male	Female	
Control	9	10	46.3
T2DM	8	9	55.1
Periodontitis	9	8	53
Diabetic periodontitis	7	7	48.5

### 3.2 T2DM patients had worse periodontal conditions

**Figure 2:**
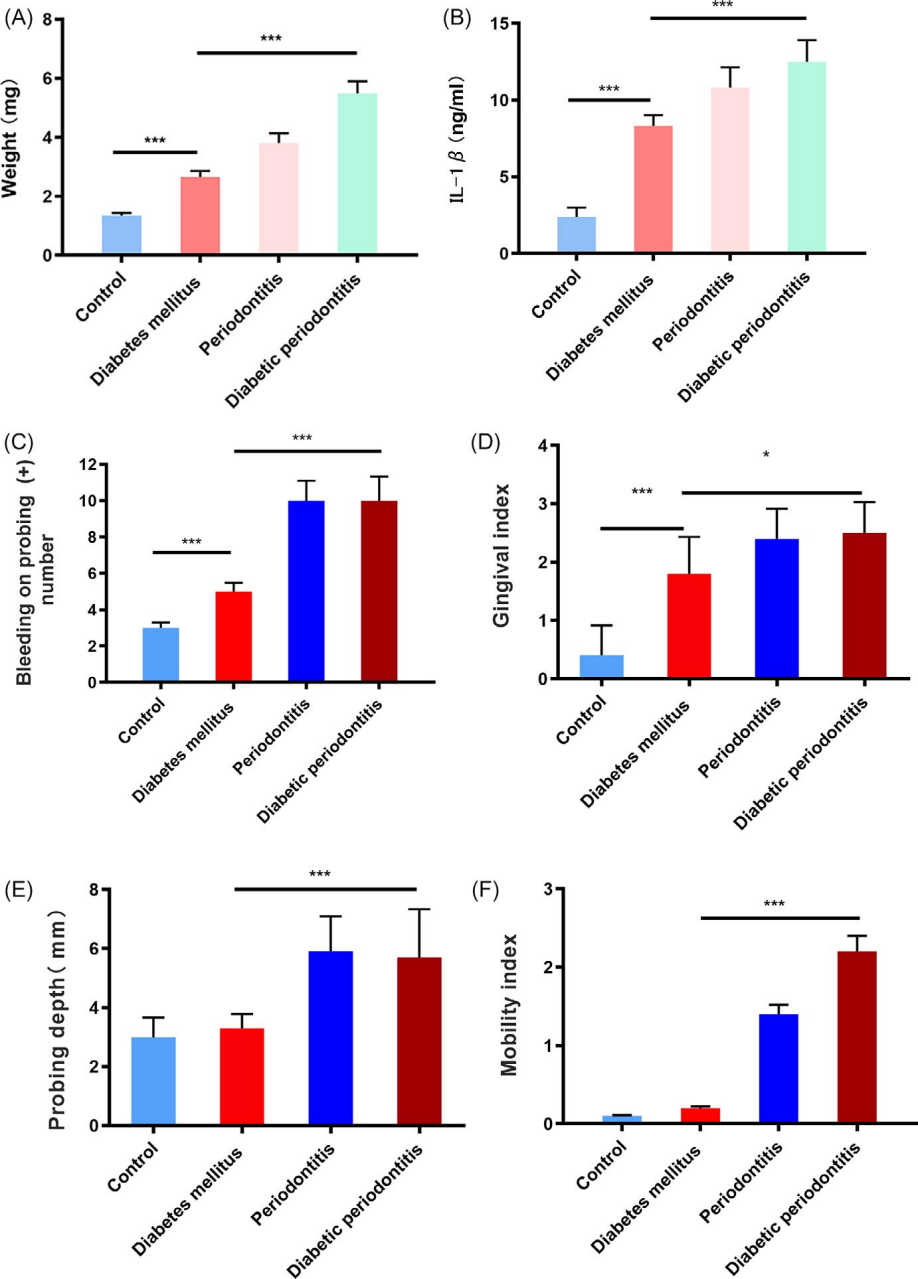
Periodontal conditions in non‐diabetic individuals, T2DM patients, patients with periodontitis and diabetic periodontitis patients. A, Weight of GCF of four groups. ***P < .001. B, Level of IL‐1β in the GCF. ***P < .001. C, Bleeding on probing of four groups. ***P < .001. D, Gingival index of four groups. *P < .05, ***P < .001. E, Probing depth of four groups. ***P < .001. F, Mobility index of four groups. ***P < .001

The weight of GCF was gained in the T2DM group compared with non‐diabetic individuals, while the weight gained even more in the periodontitis patients and diabetic periodontitis patients (Figure 2A). The level of IL‐1β in the GCF, which was the key cytokine in the development of periodontal disease, also increased in the T2DM group, periodontitis group and diabetic periodontitis group (Figure 2B). Further examination of the periodontal conditions in the four groups revealed that the BOP and GI of patients with T2DM were higher than that of non‐diabetic individuals, while they were even higher in the periodontitis patients and diabetic periodontitis patients (Figure 2C,D). No significant difference in the probing depth and tooth mobility was found between T2DM group and non‐diabetic individuals, while they were increased in the periodontitis group and diabetic periodontitis group (Figure 2E,F).

### 3.3 Increased C3 was closely related to periodontal destruction in diabetic mice

**Figure 3:**
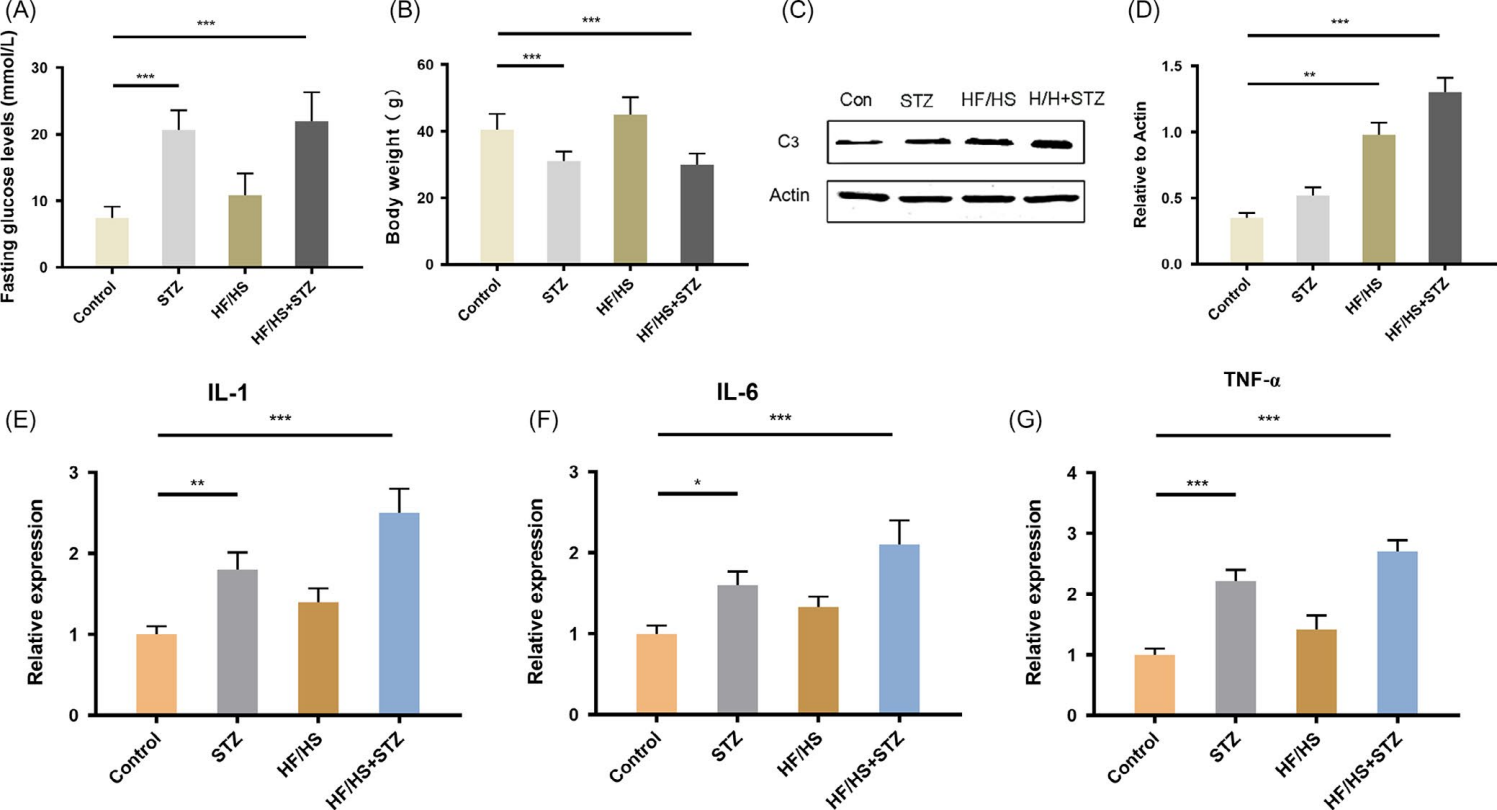
Increased C3 was closely related to periodontal destruction in diabetic mice. A, Fasting glucose levels of the mice after modelling. ***P < .001 B, Bodyweight of the mice after modelling. ***P < .001. C, C3 levels in the periodontal tissues by Western blotting. D, Quantification of Western blotting. **P < .01, ***P < .001. E, The expression of IL‐1 in gingiva by real‐time PCR. **P < .01, ***P < .001. F, The expression of IL‐6 in gingiva by real‐time PCR. *P < .05, ***P < .001. G, The expression of TNF‐α in gingiva by real‐time PCR. ***P < .001

According to Figures 1 and 2, we suspected that the increase in C3 levels of T2DM patients was closely related to the worse periodontal conditions. To prove, we established diabetic mouse model and measured the changes in bodyweight and blood glucose of the mice after modelling (Figure 3A,B). A HF/HS diet for 4 weeks without STZ injection (Diet group) would not cause elevated bodyweight and blood glucose. While basic diet with STZ injection (T1DM group) led to increased bodyweight and blood glucose as HF/HS diet with STZ injection (T2DM group). The expression of C3 in the gingival tissues of the mice was then determined by Western blotting (Figure 3C,D). The C3 levels in T2DM group were significantly higher than those of normal mice, while no significant difference was seen between normal mice and T1DM mice. We next observed inflammation of the gingival tissue in the diabetic mice (Figure 3E‐G). The expression of inflammatory cytokines including interleukin (IL)‐1, IL‐6 and tumour necrosis factor‐alpha (TNF‐α) in the gingiva of diabetic mice was significantly increased. While no significant difference was seen in the Diet group. Therefore, we may conclude that T2DM‐related C3 upregulation was closely related to the development of periodontal inflammation.

### 3.4 Deletion of C3 inhibited periodontal destruction in diabetic mice

**Figure 4:**
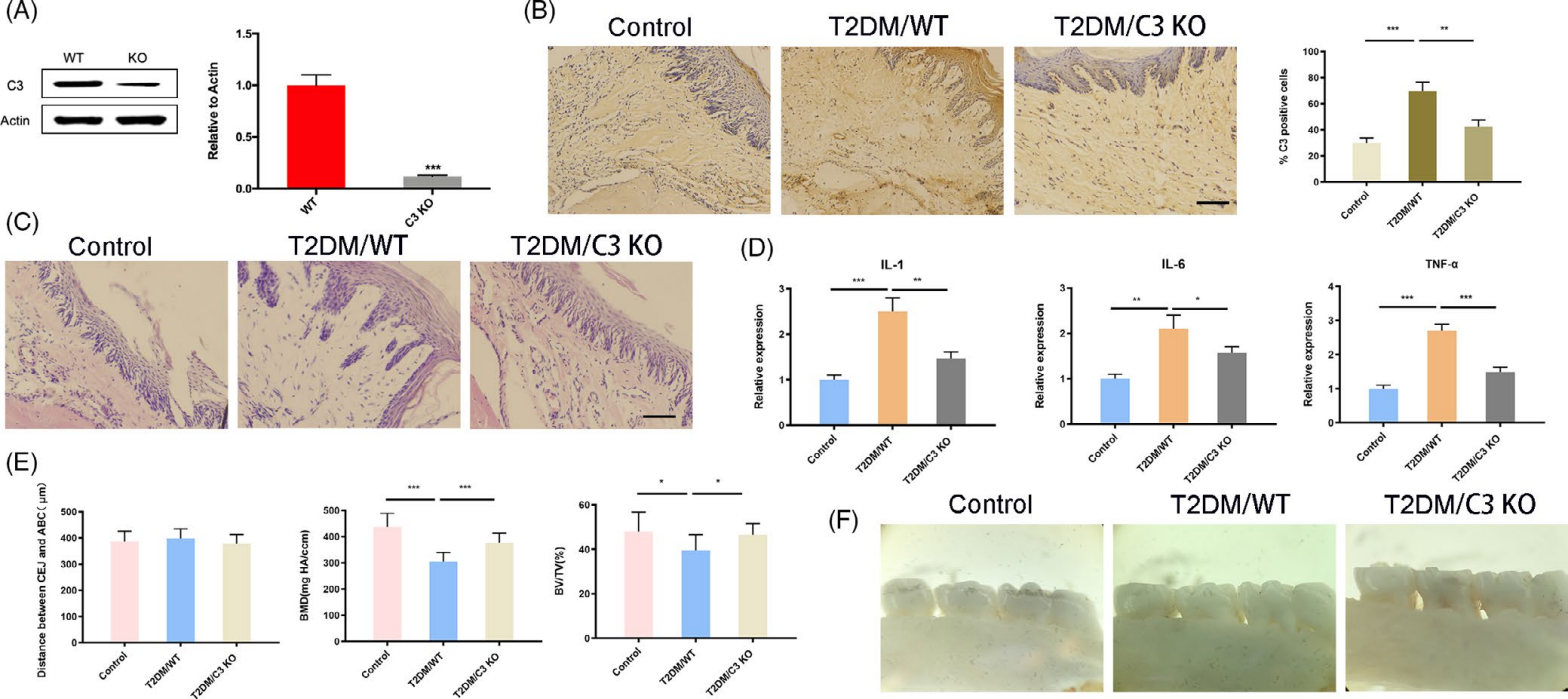
Deletion of C3 inhibited periodontal destruction in diabetic mice. A, Knockout effect of C3 in the mouse model by Western blotting. ***P < .001. B, Knockout effect of C3 in the mouse model by immunohistochemistry. Scale bar: 1 mm. **P < .01; ***P < .001. C, HE staining of the periodontal tissues after C3 deletion. Scale bar: 1 mm. D, The expression of IL‐1, IL‐6 and TNF‐α in C3 knockout mice. *P < .05; **P < .01; ***P < .001. E, CEJ‐ABC, BMD and BV/TV of the alveolar bones by micro‐CT. *P < .05; ***P < .001. F, Alveolar bone loss in three groups

To further prove that C3 is a key molecule for T2DM to promote periodontal disease, we established a C3 knockout mouse model and induced them with T2DM. The knockout effect of the mouse model was first verified in the gingival tissues by Western blotting and immunohistochemistry, showing that C3 expression was strongly inhibited (Figure 4A,B). Decreased inflammation of the gingival tissue was observed in the C3 KO mice (Figure 4C). Further investigation of the diabetic mouse model showed that the deletion of C3 significantly downregulated the inflammatory cytokines IL‐1, IL‐6 and TNF‐α compared with the non‐knockout group (Figure 4D). Micro‐CT showed that there was no difference in CEJ‐ABC among the three groups, while the deletion of C3 rescued the decreased BMD and BV/TV of the alveolar bones (Figure 4E). No significant difference was observed in the alveolar bone of C3 KO and WT mice (Figure 4F).

### 3.5 C3‐mediated polarization of macrophages was responsible for the periodontal damage

**Figure 5:**
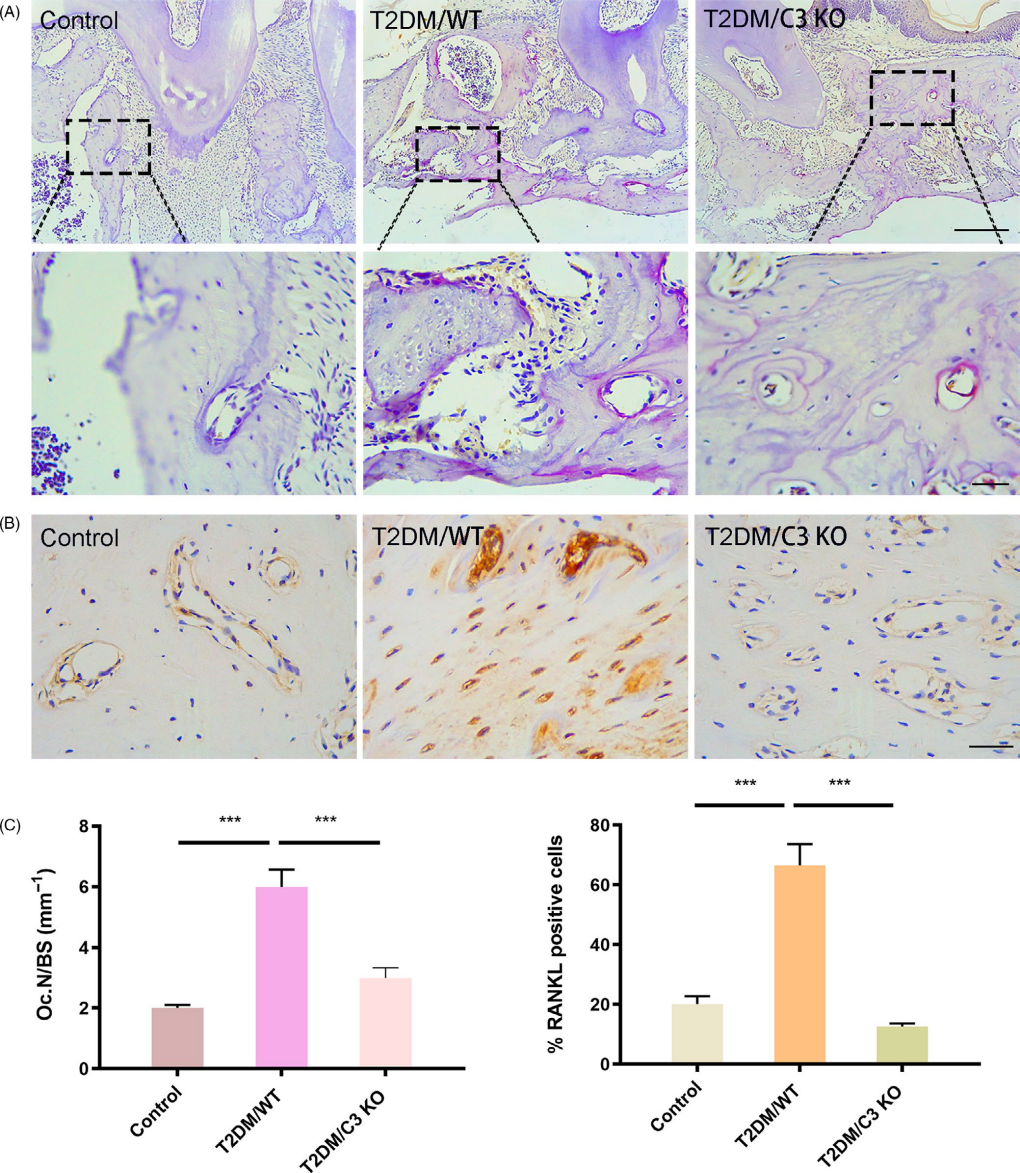
C3 was responsible for the periodontal damage in alveolar bone. A, Osteoclasts staining in the alveolar bone by trap. Scale bar: 100 μm (up) Scale bar: 50 μm (down). B, RANKL levels in the alveolar bone by immunohistochemistry. Scale bar: 50 μm. C, Quantification of osteoclasts staining and RANKL staining. ***P < .001

In this study, we also assessed osteoclasts in the alveolar bone and showed that they increased significantly in the diabetic mice, while their number in alveolar bone decreased after the deletion of C3 (Figure 5A). RANKL was an indicator of alveolar bone destruction in periodontitis. So we next evaluated RANKL expression in the alveolar bone. We found that the expression of RANKL was increased in the diabetic mice, while decreased after the deletion of C3 (Figure 5B). The quantitative results are shown in Figure 5C. We suggest that although the increase in C3 due to T2DM did not cause evident bone destruction, the increased number of osteoclasts may be the reason for the promotion of alveolar bone resorption.

**Figure 6:**
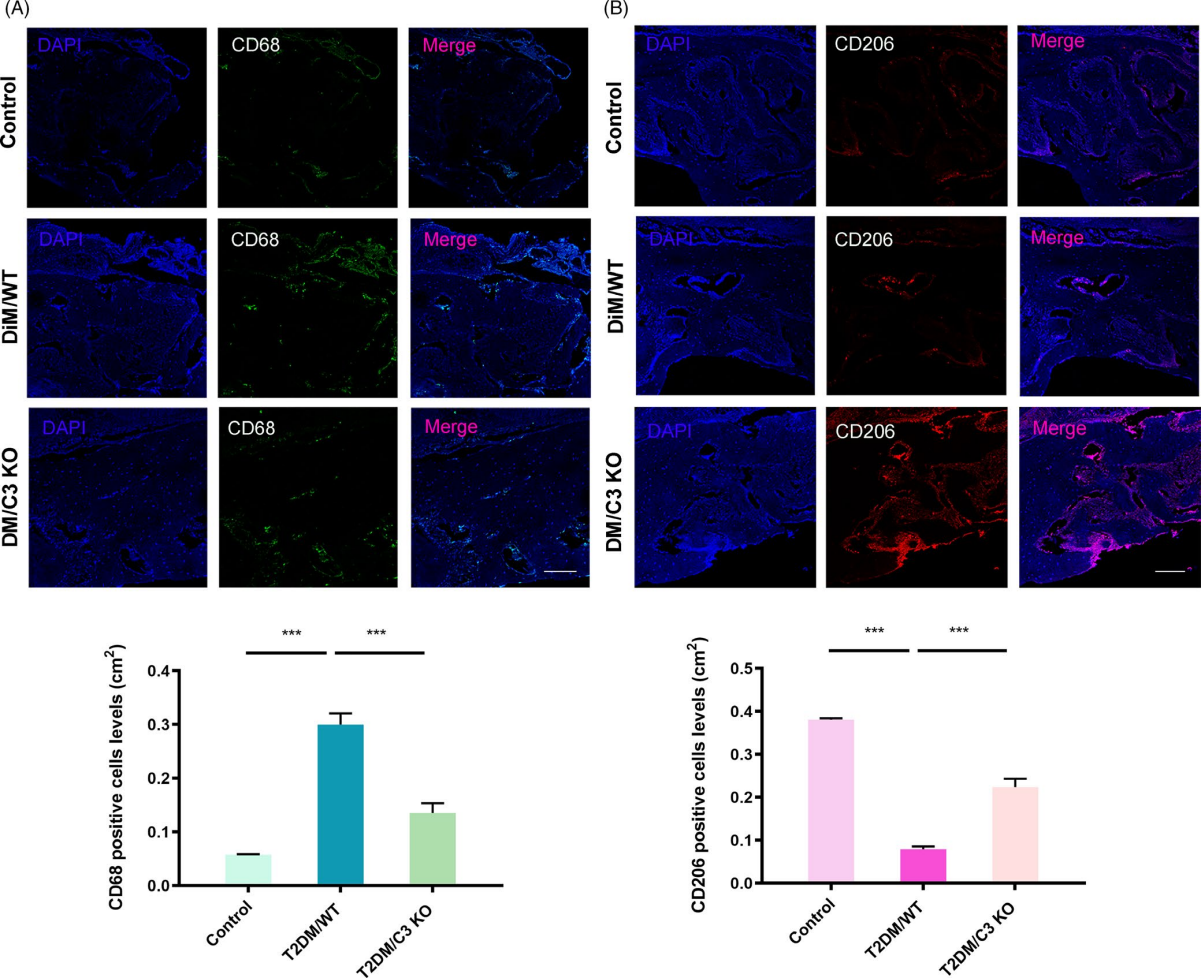
C3 promoted M1 polarization and inhibited M2 polarization of macrophages in periodontal tissues. A, The expression of CD68 (Macrophage M1 markers) in the periodontal tissues in T2DM WT/C3 KO mice by immunofluorescence. Scale bar: 200 μm. ***P < .001. B, The expression of CD206 (Macrophage M2 markers) in the periodontal tissues in T2DM WT/C3 KO mice by immunofluorescence. Scale bar: 200 μm. ***P < .001

We next investigated the mechanism of C3 regulation in the development of T2DM‐associated periodontal destruction. C3 was considered to be closely related to macrophages. The expression of C3 can affect the polarization of macrophages, promoting M1 polarization while inhibiting M2 polarization. Macrophage polarization has dual role as killers (M1) or builders (M2) in chronic inflammatory diseases, such as periodontitis. Therefore, we next examined the polarization of macrophages in the periodontal tissues. As shown in Figure 6A, M1 macrophages (CD68) in the periodontal tissues of diabetic mice increased significantly, while deletion of C3 reduced the numbers of M1 macrophages. M2 macrophages (CD206) in the periodontal tissues of diabetic mice decreased significantly, while deletion of C3 rescued the numbers of M2 macrophages (Figure 6B). These results confirmed that macrophages could be one of the C3‐targeted cells in the periodontal tissue.

## 4 DISCUSSION

In this study, we found that the increase in C3 levels of T2DM patients may be the cause of periodontal destruction. Although it did not cause obvious bone destruction, the increase in the number of osteoclasts may aggravate periodontal bone destruction. Increase in C3 caused macrophages in the periodontal tissues to polarize to M1 and inhibited the M2 polarization which may be a mechanism by which it promotes periodontitis.

Diabetes affected bacteria‐host interactions to promote inflammation and periodontal disease.^23^ The inflammatory response modified oral microbiota to render it more pathogenic. Complement system may also be involved in the process. Amplified immune response mediated by T2DM‐related C3 upregulation may lead to further bacteria‐host interactions which aggravate periodontal damage. We showed that C3 levels in the blood and GCF of T2DM patients were elevated, and so was the weight of the GCF. In healthy periodontal tissues, the secretion of GCF is limited. Increased GCF was resulted from the break of bacteria and their products from biofilm into gingival crevasse. Therefore, increased GCF is one of the main manifestations of early gingivitis and often precedes changes in clinical characteristics.^24^ When gingivitis becomes obvious, the GCF increases substantially, and inflammatory cytokine concentrations also increase.^25^ This study showed that the weight of GCF of T2DM patients was higher than that of non‐diabetic individuals. In addition, the inflammatory cytokine concentrations of T2DM patients were also elevated, suggesting that T2DM patients had a tendency for early gingival inflammation compared with non‐diabetic individuals, which may be related to the increase of C3 levels.

In the present study, we first fed mice with high fat and high sugar food for 4 weeks to induce insulin resistance, then low dose of STZ injection was followed for next 4 weeks to destruct islet tissues to establish T2DM model. Basic diet with STZ injection (T1DM) did not change the expression of C3, which was in line with previous studies.^26^ We further showed that the significant increase in C3 levels in the periodontal tissues of T2DM was closely related to periodontal inflammation, but no significant alveolar bone loss was observed. A number of functional studies used specific inhibitors of inflammatory mediators, such as TNF‐α to extenuate periodontal inflammation in diabetic models.^27^ TNF‐α inhibition reversed increased inflammation caused by diabetes. The C3 knockout in the present study also caused a decrease in the expression of inflammatory factors, such as IL‐6 and TNF‐α, thereby reducing inflammation caused by diabetes. All of which demonstrated the importance of inflammatory factors in the progress in diabetic‐related periodontal disease.

C3 can be cleaved into C3a and C3b by C3 convertase.^28^ C3a participates in the immune‐inflammatory response while C3b is inactivated by factors H and I to generate iC3b. In addition, C3b expands the effect of complement activation and generates positive feedback regulation. C3a is closely related to gingivitis and can modulate IL‐1β secretion in human monocytes.^29^ We may suggest that upregulation of C3 in T2DM patients influenced periodontal inflammation through C3a modulation to macrophage. While due to the expansion of complement activation through C3b, inflammation in periodontal tissues would develop continuously without intervention.

Periodontal inflammation is a chronic and long‐term process. Although no bone destruction was found over the short‐term, micro‐CT showed that BMD and BV/TV decreased in the diabetic mice. Trap staining showed a significant increase in the number of osteoclasts, and damage to the alveolar bone possibly appeared at subsequent time points. Since some serious complications were observed in diabetic mice, we did not extend the observation period due to ethical considerations. Deletion of C3 rescued the periodontal inflammation phenotype in diabetic mice, suggesting that the control of C3 levels in diabetic patients may reduce the risk of periodontal disease at an early stage. We found that an increase in C3 caused upregulation of the inflammatory cytokines IL‐1, IL‐6 and TNF‐α in the gingiva, inducing M1 macrophage polarization and inhibiting M2 polarization. Macrophages are very important immune cells and are closely related to the complement system.^30^, ^31^ Macrophages secrete complements that regulate the immune status.^32^ Changes in complements affect macrophages and their functions. Mutual regulation of the two is complementary and jointly regulates the occurrence and development of physiological and pathological processes. Study showed that induction of M2 macrophages prevented bone loss in periodontitis.^33^ M1 macrophages induced osteoclast activation, while M2 macrophages were involved in inflammation resolution and tissue regeneration. In the present study, T2DM upregulation of C3 induced M1 polarization and inhibited M2 polarization, which resulted in gingival inflammation and potential bone loss. The increase in osteoclasts may lead to severe alveolar bone destruction in the late stage. Therefore, we may suggest that by controlling C3 concentration at early stage of T2DM, we can prevent later periodontal destruction. C3 concentrations in GCF of diabetic patients may also be a potential indicator of T2DM‐related periodontitis.

## 5 CONCLUSION

In summary, this study showed that C3 levels were significantly increased in the blood and GCF of T2DM patients. Increased C3 levels were accompanied by periodontal destruction. Deletion of C3 extenuated gingival inflammation and rescued potential bone destruction. C3 increased M1 macrophage polarization and decreased M2 polarization, suggesting that C3 may promote the development and progression of periodontal inflammation by regulating macrophages polarization.

## CONFLICT OF INTEREST

The authors declare no conflicts of interest to the authorship and/or publication of this article.

## AUTHOR CONTRIBUTIONS

Ye Li, contributed to conception, design, data acquisition, analysis, and interpretation, drafted and critically revised the manuscript. Xinxin Wang, contributed to conception, design, data analysis and interpretation, critically revised the manuscript. Saisai Wang, Chunhui Zhu and Jing Guo contributed to data acquisition, analysis, and interpretation, critically revised the manuscript. Ke Li, contributed to conception, data acquisition and analysis, critically revised the manuscript. Ang Li, contributed to conception, design, data analysis and interpretation, drafted and critically revised the manuscript. All authors gave final approval and agreed to be accountable for all aspects of the present work.

## ETHICAL APPROVAL AND CONSENT TO PARTICIPATE

All experiments were reviewed and approved by the Ethics Committee of College of Stomatology, Xi'an Jiaotong University.

## Data Availability

The data that support the findings of this study are available from the corresponding author upon reasonable request.

## References

[1] Thomas CC , Philipson LH . Update on diabetes classification. Med Clin North Am. 2015;99(1):1‐16. 25456640 10.1016/j.mcna.2014.08.015

[2] Brunton S . Pathophysiology of type 2 diabetes: the evolution of our understanding. J Fam Pract. 2016;65(4 Suppl):supp_az_0416. 27262256

[3] Hackett RA , Steptoe A . Type 2 diabetes mellitus and psychological stress – a modifiable risk factor. Nat Rev Endocrinol. 2017;13(9):547‐560. 28664919 10.1038/nrendo.2017.64

[4] Loe H . Periodontal disease. The sixth complication of diabetes mellitus. Diabetes Care. 1993;16(1):329‐334. 8422804

[5] Kudiyirickal MG , Pappachan JM . Diabetes mellitus and oral health. Endocrine. 2015;49(1):27‐34. 25487035 10.1007/s12020-014-0496-3

[6] Lalla E , Papapanou PN . Diabetes mellitus and periodontitis: a tale of two common interrelated diseases. Nat Rev Endocrinol. 2011;7(12):738‐748. 21709707 10.1038/nrendo.2011.106

[7] Zhou X , Zhang W , Liu X , Zhang W , Li Y . Interrelationship between diabetes and periodontitis: role of hyperlipidemia. Arch Oral Biol. 2015;60(4):667‐674. 25443979 10.1016/j.archoralbio.2014.11.008

[8] Ghosh P , Sahoo R , Vaidya A , Chorev M , Halperin JA . Role of complement and complement regulatory proteins in the complications of diabetes. Endocr Rev. 2015;36(3):272‐288. 25859860 10.1210/er.2014-1099 PMC4446516

[9] Flyvbjerg A . The role of the complement system in diabetic nephropathy. Nat Rev Nephrol. 2017;13(5):311‐318. 28262777 10.1038/nrneph.2017.31

[10] Ostergaard J , Hansen TK , Thiel S , Flyvbjerg A . Complement activation and diabetic vascular complications. Clin Chim Acta. 2005;361(122):10‐19. 15996650 10.1016/j.cccn.2005.04.028

[11] Xu H , Chen M . Diabetic retinopathy and dysregulated innate immunity. Vision Res. 2017;139:39‐46. 28571700 10.1016/j.visres.2017.04.013

[12] Nonaka M . Evolution of the complement system. Curr Opin Immunol. 2001;13(1):69‐73. 11154920 10.1016/s0952-7915(00)00184-9

[13] Ricklin D , Reis ES , Mastellos DC , Gros P , Lambris JD . Complement component C3 – The "Swiss Army Knife" of innate immunity and host defense. Immunol Rev. 2016;274(1):33‐58. 27782325 10.1111/imr.12500 PMC5427221

[14] Fujita T , Hemmi S , Kajiwara M , et al. Complement‐mediated chronic inflammation is associated with diabetic microvascular complication. Diabetes Metab Res Rev. 2013;29(3):220‐226. 23280928 10.1002/dmrr.2380

[15] Barbu A , Hamad OA , Lind L , Ekdahl KN , Nilsson B . The role of complement factor C3 in lipid metabolism. Mol Immunol. 2015;67(1):101‐107. 25746915 10.1016/j.molimm.2015.02.027

[16] Onat A , Can G , Rezvani R , Cianflone K . Complement C3 and cleavage products in cardiometabolic risk. Clin Chim Acta. 2011;412(13‐14):1171‐1179. 21419112 10.1016/j.cca.2011.03.005

[17] Ursini F , Abenavoli L . The emerging role of complement C3 as a biomarker of insulin resistance and cardiometabolic diseases: preclinical and clinical evidence. Rev Recent Clin Trials. 2018;13(1):61‐68. 29189176 10.2174/1574887112666171128134552

[18] Hajishengallis G , Lambris JD . Complement and dysbiosis in periodontal disease. Immunobiology. 2012;217(11):1111‐1116. 22964237 10.1016/j.imbio.2012.07.007 PMC3439646

[19] Maekawa T , Abe T , Hajishengallis E , et al. Genetic and intervention studies implicating complement C3 as a major target for the treatment of periodontitis. J Immunol. 2014;192(12):6020‐6027. 24808362 10.4049/jimmunol.1400569 PMC4078411

[20] American Diabetes A . 2. classification and diagnosis of diabetes: standards of medical care in diabetes‐2018. Diabetes Care. 2018;41(Suppl 1):S13‐S27. 29222373 10.2337/dc18-S002

[21] Furman BL . Streptozotocin‐induced diabetic models in mice and rats. Curr Protoc Pharmacol. 2015;70(1):41‐45. 10.1002/0471141755.ph0547s70 26331889

[22] Li YE , Wang X , Ren J , et al. Mandible exosomal ssc‐mir‐133b regulates tooth development in miniature swine via endogenous apoptosis. Bone Res. 2018;6:28. 30210900 10.1038/s41413-018-0028-5 PMC6131536

[23] Graves DT , Ding Z , Yang Y . The impact of diabetes on periodontal diseases. Periodontol 2000. 2020;82(1):214‐224. 31850631 10.1111/prd.12318

[24] Huynh AHS , Veith PD , McGregor NR , et al. Gingival crevicular fluid proteomes in health, gingivitis and chronic periodontitis. J Periodontal Res. 2015;50(5):637‐649. 25439677 10.1111/jre.12244

[25] Stadler AF , Angst PD , Arce RM , Gomes SC , Oppermann RV , Susin C . Gingival crevicular fluid levels of cytokines/chemokines in chronic periodontitis: a meta‐analysis. J Clin Periodontol. 2016;43(9):727‐745. 27027257 10.1111/jcpe.12557

[26] Stanekova D , Niks M , Buc M , Starsia Z , Michalkova . Genetic polymorphism of factor B (Bf) and C3 component of complement in type 1 (insulin‐dependent) diabetes mellitus: BFQO allele observed in a diabetic child. Folia Biol (Praha). 1993;39(3):117‐123. 8157131

[27] Pacios S , Kang J , Galicia J , et al. Diabetes aggravates periodontitis by limiting repair through enhanced inflammation. FASEB J. 2012;26(4):1423‐1430. 22179526 10.1096/fj.11-196279 PMC3316902

[28] Hajishengallis G , Kajikawa T , Hajishengallis E , et al. Complement‐dependent mechanisms and interventions in periodontal disease. Front Immunol. 2019;10:406. 30915073 10.3389/fimmu.2019.00406 PMC6422998

[29] Asgari E , Le Friec G , Yamamoto H , et al. C3a modulates IL‐1beta secretion in human monocytes by regulating ATP efflux and subsequent NLRP3 inflammasome activation. Blood. 2013;122(20):3473‐3481. 23878142 10.1182/blood-2013-05-502229

[30] Roos D . Complement and phagocytes – a complicated interaction. Mol Immunol. 2015;68(1):31‐34. 26597203 10.1016/j.molimm.2015.05.001

[31] van Lookeren CM , Wiesmann C , Brown EJ . Macrophage complement receptors and pathogen clearance. Cell Microbiol. 2007;9(9):2095‐2102. 17590164 10.1111/j.1462-5822.2007.00981.x

[32] Lubbers R , van Essen MF , van Kooten C , Trouw LA . Production of complement components by cells of the immune system. Clin Exp Immunol. 2017;188(2):183‐194. 28249350 10.1111/cei.12952 PMC5383442

[33] Zhuang Z , Yoshizawa‐Smith S , Glowacki A , et al. Induction of M2 macrophages prevents bone loss in murine periodontitis models. J Dent Res. 2019;98(2):200‐208. 30392438 10.1177/0022034518805984 PMC6761736

