# Correction to “Complement 3 Mediates Periodontal Destruction in Patients With Type 2 Diabetes by Regulating Macrophage Polarization in Periodontal Tissues”

**DOI:** 10.1111/cpr.70128

**Published:** 2026-03-17

Li, Y., X. Wang, S. Wang, et al. 2020. “Complement 3 Mediates Periodontal Destruction in Patients With Type 2 Diabetes by Regulating Macrophage Polarization in Periodontal Tissues.” *Cell Proliferation* 53, no. 10: e12886.

**FIGURE 6:**
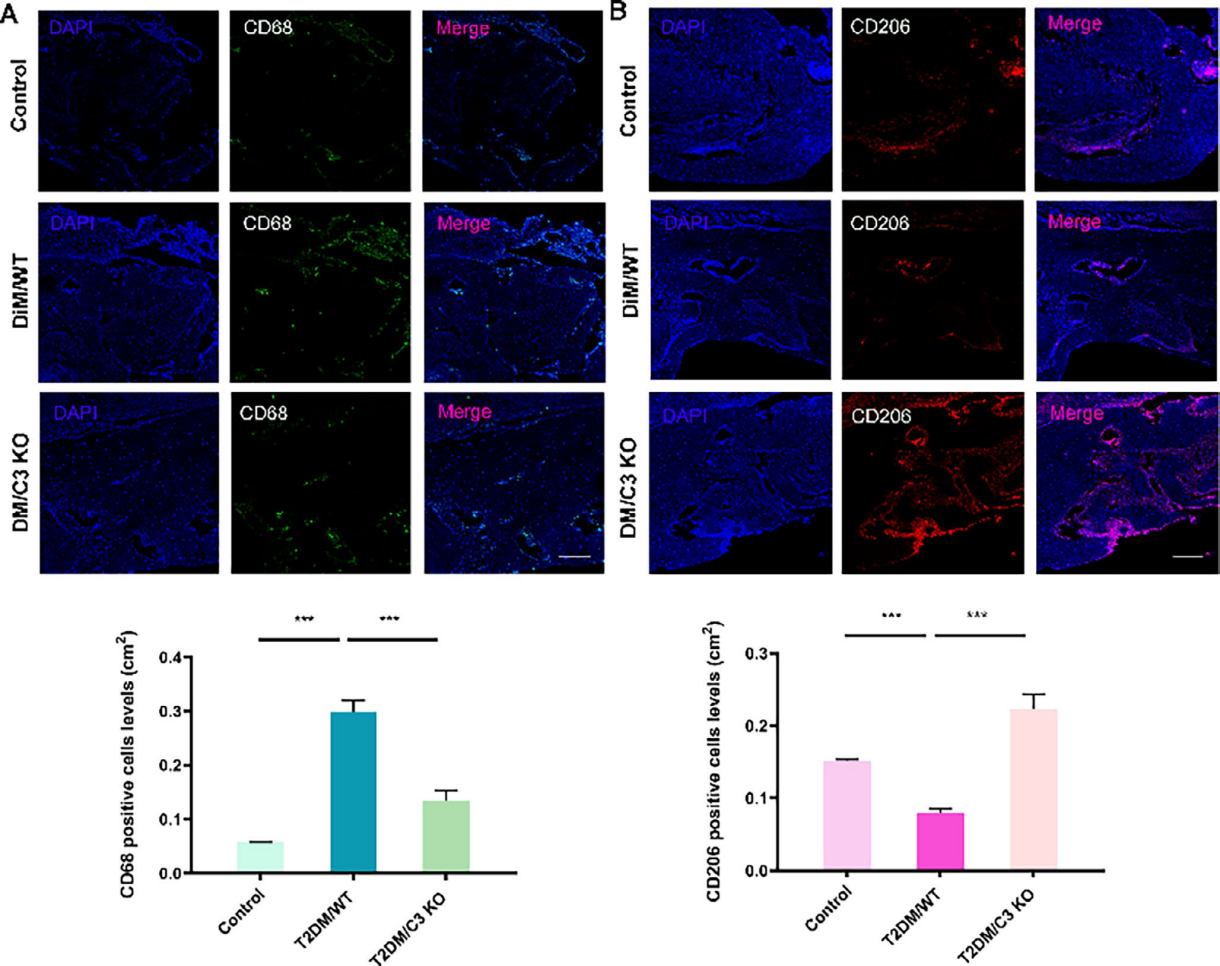
C3 promoted M1 polarization and inhibited M2 polarization of macrophages in periodontal tissues. (A) The expression of CD68 (Macrophage M1 markers) in the periodontal tissues in T2DM WT/C3 KO mice by immunofluorescence. Scale bar: 200 μm. ***p < 0.001. (B) The expression of CD206 (Macrophage M2 markers) in the periodontal tissues in T2DM WT/C3 KO mice by immunofluorescence. Scale bar: 200 μm. ***p < 0.001.

In the originally published article, the “ETHICAL APPROVAL AND CONSENT TO PARTICIPATE” section did not include the ethical approval numbers. The section should read as follows:

All experiments were reviewed and approved by the Ethics Committee of the College of Stomatology, Xi'an Jiaotong University (XJKQ‐2018‐36 for human subjects experiments and XJTU‐2019‐412 for mouse experiments).

Due to an oversight during the final stage of figure preparation, the representative images for the Control group in Figure 6 were incorrectly inserted. The correct Figure 6 is provided below:

We apologize for these errors.

